# A Scientometric Analysis and Visualization Discovery of Enhanced Recovery After Surgery

**DOI:** 10.3389/fsurg.2022.894083

**Published:** 2022-06-08

**Authors:** Mingjie Zhang, Xiaoxue Wang, Xueting Chen, Zixuan Song, Yuting Wang, Yangzi Zhou, Dandan Zhang

**Affiliations:** ^1^ Department of Surgery, Shengjing Hospital of China Medical University, Shenyang, China; ^2^ Department of Health Management, Shengjing Hospital of China Medical University, Shenyang, China; ^3^ Department of Obstetrics and Gynecology, Shengjing Hospital of China Medical University, Shenyang, China

**Keywords:** enhanced recovery after surgery, fast-track surgery, bibliometrics, bibliometrix, citespace

## Abstract

**Background:**

Enhanced recovery after surgery (ERAS), a new clinical surgical concept, has been applied in many surgical disciplines with good clinical results for the past 20 years. Bibliometric analysis is an effective method to quantitatively evaluate the academic productivity. This report aimed to perform a scientometric analysis of the ERAS research status and research hotspots.

**Methods:**

Comprehensive scientific mapping analysis of a wide range of literature metadata using the scientometric tools, including the Bibliometrix R Package, Biblioshiny, and CiteSpace. Data were retrieved from the Web of Science Core Collection database of original articles from 2001 to 2020. Specific indicators and maps were analyzed to show the co-authorship, co-institute, co-country, co-citation, and international cooperation. Automatic literature screening, unsupervised cluster filtering, and topic cluster identification methods were used to display the conceptual framework and thematic evolution.

**Results:**

A total of 1,403 research projects drafted by 6,966 authors and published in 413 sources were found. There was an exponential growth in the number of publications on ERAS. There were 709 collaborations between authors from different countries, and the US, China, and the UK had the greatest number of publications. The WORLD JOURNAL OF SURGERY, located in Bradford’s Law 1, had the highest number of published articles (*n* = 1,276; total citations = 3,193). CiteSpace network analysis revealed 15 highly correlated cluster ERAS studies, and the earliest study was on colonic surgery, and ERAS was recently applied in cardiac surgery. The etiology of ERAS is constantly evolving, with surgery and length of hospital as the main topics. Meta-analyses and perioperative care have tended to decline.

**Conclusion:**

This is the first scientometric analysis of ERAS to provide descriptive quantitative indicators. This can provide a better understanding of how the field has evolved over the past 20 years, help identify research trends, and provide insights and research directions for academic researchers, policymakers, and medical practitioners who want to collaborate in these areas in the future.

## Introduction

Enhanced recovery after surgery (ERAS) was first proposed by Kehlet et al. at the University of Copenhagen in Denmark in the late 1990s and has been applied clinically (1, 2). ERAS was initially used primarily in Europe and North America to study the effects of surgical stress response on open colorectal surgery in terms of rapid recovery. ERAS represents the idea of synergy through a combination of effective measures, with the central aim of reducing trauma and stress. ERAS is the best result of multidisciplinary collaboration that perfectly blends the latest research findings from surgery, anesthesia, and nursing into an integrated innovative concept that represents an optimized clinical pathway. This optimized clinical pathway involves the whole process of patient diagnosis and treatment, emphasis on the patient-centered concept. The implementation of the ERAS pathway can improve perioperative safety and the satisfaction in surgical patients, shorten the postoperative hospital stay, and help reduce the incidence of postoperative complications (3). Using the ERAS, patients can be identified, compartmentalized, and accommodated at every step throughout the perioperative period to facilitate an effective and safe process from preoperative evaluation through discharge to recovery. The advantages of ERAS have been recognized by operators and specialists worldwide, and its application in the medical field has been actively promoted. The ERAS Society has developed numerous perioperative guidelines for ERAS for various specialties and disciplines since 2005 (4–11).

The postoperative rehabilitation of patients undergoing surgery is affected by various factors, such as stress response, pain, and postoperative intestinal paralysis (12). stress response after surgery is a physiological and pathological process in the body, including changes in the nerve, endocrine, metabolic, and immune functions. Similarly, pain can adversely affect patient recovery. Postoperative intestinal paralysis aggravates postoperative discomfort, especially in patients undergoing abdominal surgery, affecting oral feeding and delaying the recovery of patients. Therefore, the combination of new techniques in anesthesiology, pain control, and surgical methods with the improvement of the traditional postoperative nursing methods can reduce the postoperative stress reaction, incidence of postoperative complications and mortality, postoperative hospital stay, and hospitalization cost. This finding is consistent with the concept of minimally invasive surgery. ERAS generally includes the following: (1) Preoperative patient education. (2) better anesthesia, analgesia, and surgical techniques can reduce surgical stress, pain, and discomfort. (3) enhanced postoperative rehabilitation including early ambulation and enteral nutrition. Several surgical studies have shown that ERAS can significantly shorten the postoperative hospitalization time, reduce hospitalization costs, and maximize the use of limited hospital resources without increasing the incidence of complications and mortality, which will become the trend of surgery (13, 14).

ERAS has been applied and supplemented for 20 years since it was first proposed in 2001, and is now being developed in a more refined direction. Its research direction is not limited to the surgical field, and future research directions and current research hotspots need to be clarified. Bibliometric analysis is the application of statistical and mathematical tools to books and media communications (15). Bibliometric analysis is a transparent, systematic, and repeatable review process that significantly improves the quality of literature review. This provides a means of mapping research fields and influential work without subjective bias.

This study aimed to identify research flows and topics by analyzing the citation dynamics in ERAS studies from to 2001–2020 to measure their impact on the scientific community (qualitative indicators). These topics and streams of research can guide scholars to find directions for future research and to find answers to current questions.

## Materials & Method

### Study Design/Ethics Statement

**Figure 1:**
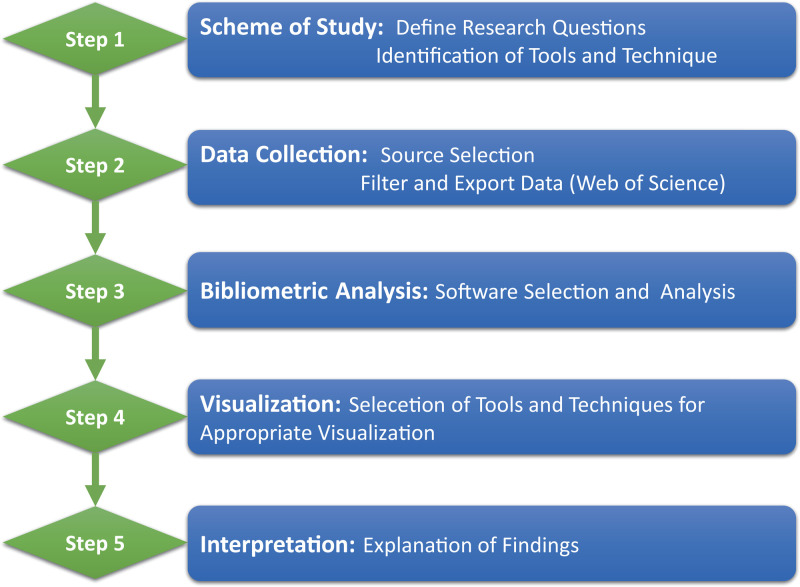
The Procedure of Bibliometric Analysis.

This was a bibliometric network study using metadata from the Web of Science Core Collection on July 12, 2021. This is described in accordance with the STROBE guidelines (16). This was not a human-focused study and, as such, neither the institutional review board approval nor informed consent was required. This study was divided into five steps, known as the document metering workflow proposed by Zupic and Ater (17). Figure 1 represents the five steps used to complete the bibliometric analysis of ERAS.

### Data Sources

The original data for this article were obtained from the Web of Science core collection database, including SCI-EXPAND and SSCI. This following titles were used for the selection: “enhanced recovery after surgery” or “enhanced postsurgical recovery” or “enhanced recovery program” or “enhanced recovery pathways” or “accelerated rehabilitation” or “fast track surgery.” The publication date was restricted from January 1, 2001 to December 31, 2020. The manuscript type was “article” and the target results were filtered. The file format recorded content was set to “plain text” and was exported as “full record”.

### Analysis Tool

Bibliometrix is an R language based bibliometric software with multiple toolkits for full-process bibliometrics and visual presentation of scientific documents, which was developed by Aria (18). Based on the data exported from the WOS database and then completed by the team researchers (XX Wang and ZX Song), the bibliometric analysis was conducted using the R software version 3.6.2 (R Foundation for Statistical Computing, Vienna, Austria; http://www.r-project.org) through the Bibliometrix R package.

CiteSpace is a visual tool for bibliometric analysis developed by Chaomei Chen, based on the Java platform (19). As an interactive analysis tool, it combines bibliometrics and data-mining algorithms to complete scientific mapping through the visualization of results. CiteSpace can be used to analyze cooperative networks and co-citations (20). Version 5.8.3 was used in this study for the analysis of the co-authorship, co-institute, co-country, and document co-citation of the articles published from 2001 to 2020. The time slice was two years, and the selection criterion was the first 50% of each time period. In collaborative networks, the size of the circles represents the number of studies published; the shorter the distance between the circles, the greater the collaboration between the two authors/institutions/countries. The blue-purple nodes represented earlier studies, whereas the yellow-red nodes represented more recent studies. In the co-citation analysis, the size of the nodes represented the frequency of citations, nodes with different colors represented different years, the line between the nodes represented the relationship between the co-citations, and the thickness of the line represented the strength of the relationship. The color corresponds to the time of the node’s first co-citation, and the color from cold to warm represented early to recent co-citation. The thickness of the tree-ring was proportional to the number of citations in a given time zone. Modularity (Q) and weighted mean silhouette (S) were the two indicators used to evaluate clustering in the co-citation analysis. A Q value >0.3 meant very important network and an S value >0.5 meant reasonable clustering results.

### Measurement

Descriptive analysis was used to explain the core sources, authors, countries, publications, and affiliations of publications. Price’s law was used to assess whether the growing trend in the ERAS was scientific. Simultaneously, we identified the core sources using the Bradford’s law (21). According to the Bradford’s law, the data was divided into three regions. Zone 1 was highly productive and was considered a nuclear zone.

The author/publication-level metrics, such as the h-, m-, and g indices were determined (22, 23). In addition, we used keyword plus for the analysis, which was provided by the database to describe the knowledge structure of the study more concisely and standardized. The core research areas and key themes were essential for determining the direction of future research, therefore, thematic maps and thematic evolution were used.

In the thematic maps, each quadrant could be separated by centrality and density to form a two-dimensional graph. Centrality was the importance of a topic in the research field, and the density was used to measure the development of the topic. Quadrant I, located in the upper-right quadrant, named motor themes, suggested that the themes of the region have developed and formed important pillars that shape the field of research. Quadrant II, located in the upper left quadrant, named niche themes, reflected highly developed but isolated themes. Quadrant III, located in the lower-left quadrant and named emerging or declining themes, suggested weak development and marginalization of the research field. Quadrant IV, located in the lower-right quadrant, was named as basic themes. Although these topics are less developed, they are important to the field of study.

## Results

### Overall Publication Performance and Growth Rate

Table 1 presents the descriptive features of the ERAS literature. A total of 1,403 studies were selected according to the search strategy. We identified 1,320 articles and 81 articles and proceedings papers. A total of 2,098 keywords plus 1,991 author keywords were used. Furthermore, 6,966 authors wrote the documents; among them, only 26 articles were written by one author. The collaboration index was 5.07, which showed the highly collaborative nature of ERAS publications. The document-per-author ratio was 0.201, implying that, on average, approximately five authors wrote a document.

**Table 1:** Descriptive characteristics of ERAS literature.

Description	Results
Main information about data	
Timespan	2001:2021
Sources (Journals, Books, etc)	413
Documents	1,403
Average years from publication	5.27
Average citations per documents	26.72
Average citations per year per doc	3.939
References	21,902
Document types	
article	1,320
article; proceedings paper	81
Document contents	
Keywords Plus (ID)	2,098
Author’s Keywords (DE)	1,991
Authors	
Authors	6,966
Author Appearances	9,558
Authors of single-authored documents	26
Authors of multi-authored documents	6,940
Authors collaboration	
Single-authored documents	33
Documents per Author	0.201
Authors per Document	4.97
Co-Authors per Documents	6.82
Collaboration Index	5.07

**Figure 2:**
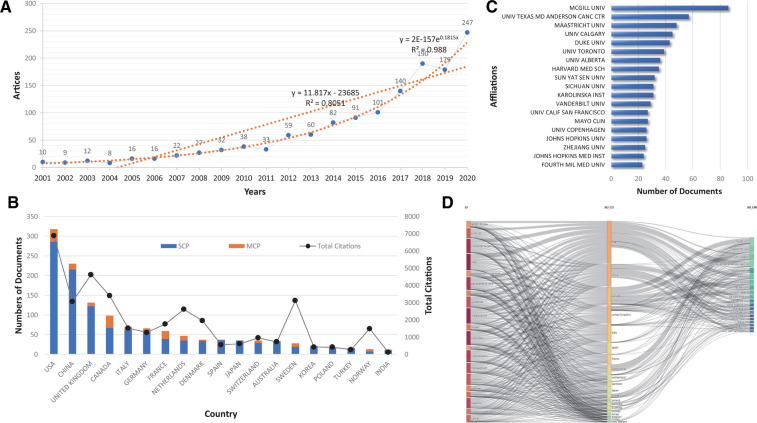
(**A**) Growth of scientific production on ERAS from 2001 to 2020. A linear adjustment of the data and an adjustment to the exponential curve were made, in order to assess whether the production fulfilled the Price’s Law. Linear adjustment: *y* = 11.8176x – 23685, *R*^2^ = 0.8051; Exponential adjustment: *y* = 2E-157e0.1815x, *R*^2^ = 0.9804. (**B**) The countries of the top 20 most relevant corresponding authors of the articles on ERAS. MCP, Multi-country publications; SCP, simple-country publications. (**C**) Top 20 of the Most Relevant Affiliations. (**D**) R Studio - Three-fields plot: left – keywords plus from the data records, middle – countries, right – authors affiliations.

By summarizing the number of papers published over the years, Figure 2A shows the overall trend of ERAS studies published worldwide from 2001 to 2020. ERAS research has an overall upward trend, and according to the curves analyzed from the data, it was found to be more suitable for exponential adjustment than linear adjustment, thus satisfying the Price’s law. The correlation coefficient (r) after mathematical adjustment of the exponential curve was 0.9804, while the linear adjustment of the measured values, R, was 0.8051. Therefore, the percentage of unexplained variation was 19.49%.

Figure 2B shows three sets of data: simple-country authors, multicountry authors, and the citation rate of each country. The USA was at the top with several publications, China was ranked second, and the UK was ranked third, but concerning citations, China’s total citations were worse than those of the UK. In contrast, although Sweden has a relatively small number of publications, it was at the top four the most cited countries, after the USA, the UK, and Canada. The country with the highest international cooperation was the USA, followed by Canada and the France. The most relevant affiliations are reported in Figure 2C. The McGill University was the first, and provided a strong basis for ERAS. The University of Texas MD Anderson Cancer Center and Maastricht University were the second and third affiliations of most publications, respectively.

In addition to the annual production, major topics, locations, and affiliations of ERAS-related publications were viewed. Figure 2D shows a threefold analysis of the ERAS publications, with keywords plus on the left, affiliations on the right, and relevant countries in the middle. The chart shows that the USA was working with most of the top agencies to focus on ERAS-related topics. In addition, China, Canada, and the UK have made significant contributions to ERAS science topics. Issues related to outcomes and care are the most widely studied in most countries.

### Core Journals, Core Journal Articles and Core Words of ERAS

**Figure 3:**
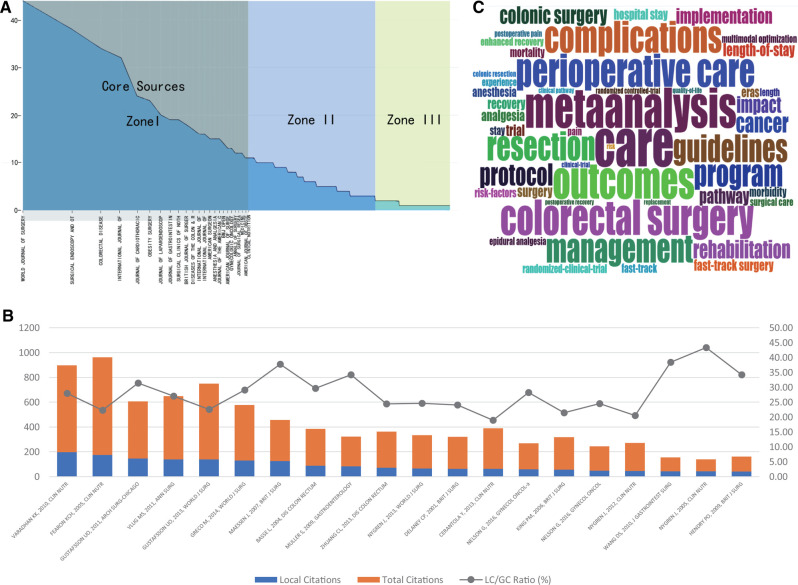
(**A**) Journal Rankings According to the Bradford Law. (**B**) Top 20 of the Most Globally Cited Article and their located cited. (**C**) Word cloud based on the most frequent words (Keywords Plus) used in the articles on ERAS.

We used source impact and Bradford’s Law to find publications on ERAS in the core journals in the scientific literature. Table 2 ranks the articles by h, M, G-index, total citations (TC), net output (NP), and pub year of publication (PYstart), and represents the Bradford’s Law (Figure 3A), which divides journals into three regions. We found that 24 of the 413 journals were in core zone 1, and the top 24 journals were the core publishing sources for corona literature in the social sciences.

**Table 2:** Zone 1 journals according Bradford’s law and the source impact.

Element	Source impact							Bradford’s law			
	Articles	h_index	g_index	m_index	TC	NP	PY_start	Rank	Freq	cumFreq	Zone
World journal of surgery	1,276	26	44	1.857143	3,193	44	2008	1	44	44	Zone 1
Surgical endoscopy and other interventional techniques	714	15	24	1.363636	666	37	2011	2	38	82	Zone 1
Colorectal disease	792	18	31	1.125	990	33	2006	3	34	116	Zone 1
International journal of colorectal disease	480	16	26	1.066667	693	31	2007	4	32	148	Zone 1
Journal of cardiothoracic and vascular anesthesia	260	16	22	0.761905	513	23	2001	5	24	172	Zone 1
Obesity surgery	398	13	22	0.8125	517	22	2006	6	23	195	Zone 1
Journal of laparoendoscopic & advanced surgical techniques	73	9	15	0.692308	255	17	2009	7	20	215	Zone 1
Journal of gastrointestinal surgery	530	14	18	0.933333	706	18	2007	8	19	234	Zone 1
Surgical clinics of North America	68	9	13	1	192	19	2013	9	19	253	Zone 1
British journal of surgery	2,355	17	18	0.809524	1,912	18	2001	10	18	271	Zone 1
Diseases of the colon & rectum	951	13	17	0.722222	1,171	17	2004	11	17	288	Zone 1
International journal of clinical and experimental medicine	20	1	2	0.142857	11	6	2015	12	16	304	Zone 1
International journal of surgery	253	11	16	1.375	383	16	2014	13	16	320	Zone 1
American surgeon	91	6	11	0.3	127	13	2002	14	15	335	Zone 1
Anesthesia and analgesia	1,102	11	15	0.52381	680	15	2001	15	15	350	Zone 1
Journal of the american college of surgeons	694	12	15	1.333333	645	15	2013	16	15	365	Zone 1
BMJ open	94	10	14	1	311	14	2012	17	14	379	Zone 1
American journal of surgery	474	7	13	0.538462	202	13	2009	18	13	392	Zone 1
Gynecologic oncology	385	9	13	1.5	601	13	2016	19	13	405	Zone 1
Annals of surgery	2,424	10	12	0.5	1,291	12	2002	20	12	417	Zone 1
Journal of surgical research	150	7	11	0.538462	172	11	2009	21	12	429	Zone 1
Medicine	98	7	11	1.166667	133	11	2016	22	12	441	Zone 1
American journal of obstetrics and gynecology	173	9	11	2.25	395	11	2018	23	11	452	Zone 1
Clinical nutrition	1,031	10	11	0.588235	3,219	11	2005	24	11	463	Zone 1

Table 3 lists the top 20 most-cited articles in the database along with their global citations. Figure 3B shows the local and global citations for highly cited papers. Table 4 provides the most frequent words used in the ERAS literature, which is divided into four parts: keywords plus, authors’ keywords, abstracts, and titles. Figure 3C shows the word cloud created using keyword plus. Words with a high frequency in the literature were larger in size.

**Table 3:** Top 20 most cited articles.

Paper	Most local cited				Most global cited			
	Local citations	Normalized local citations	Normalized global citations	Total citations	TC per year	Normalized TC	LC/GC ratio (%)	Year
The enhanced recovery after surgery (ERAS) pathway for patients undergoing major elective open colorectal surgery: a meta-analysis of randomized controlled trials	196	16.44	12.87	701	58.4167	12.8686	27.96	2010
Enhanced recovery after surgery: a consensus review of clinical care for patients undergoing colonic resection	175	11.72	10.48	787	46.2941	10.4846	22.24	2005
Adherence to the enhanced recovery after surgery protocol and outcomes after colorectal cancer surgery	145	10.22	7.83	462	42	7.8345	31.39	2011
Laparoscopy in combination with fast track multimodal management is the best perioperative strategy in patients undergoing colonic surgery: a randomized clinical trial (LAFA-study)	138	9.73	8.67	511	46.4545	8.6655	27.01	2011
Guidelines for perioperative care in elective colonic surgery: Enhanced Recovery After Surgery (ERAS(®)) Society recommendations	138	12.12	10.15	611	67.8889	10.1523	22.59	2013
Enhanced recovery program in colorectal surgery: a meta-analysis of randomized controlled trials	130	15.23	11.71	447	55.875	11.7105	29.08	2014
A protocol is not enough to implement an enhanced recovery programme for colorectal resection	125	10.11	5.33	331	22.0667	5.327	37.76	2007
Colonic surgery with accelerated rehabilitation or conventional care	88	5.46	3.56	297	16.5	3.5569	29.63	2004
A fast-track program reduces complications and length of hospital stay after open colonic surgery	82	7.09	5.13	240	18.4615	5.1268	34.17	2009
Enhanced recovery after surgery programs versus traditional care for colorectal surgery: a meta-analysis of randomized controlled trials	71	6.24	4.84	291	32.3333	4.8352	24.40	2013
Guidelines for perioperative care in elective rectal/pelvic surgery: Enhanced Recovery After Surgery (ERAS(®)) Society recommendations	66	5.80	4.45	268	29.7778	4.4531	24.63	2013
‘Fast track’ postoperative management protocol for patients with high co-morbidity undergoing complex abdominal and pelvic colorectal surgery	62	6.89	4.34	258	12.2857	4.3361	24.03	2001
Guidelines for perioperative care after radical cystectomy for bladder cancer: Enhanced Recovery After Surgery (ERAS(®)) society recommendations	62	5.45	5.45	328	36.4444	5.45	18.90	2013
Guidelines for pre- and intra-operative care in gynecologic/oncology surgery: Enhanced Recovery After Surgery (ERAS®) Society recommendations–Part I	59	10.44	8.00	209	34.8333	8.0019	28.23	2016
Randomized clinical trial comparing laparoscopic and open surgery for colorectal cancer within an enhanced recovery programme	56	5.37	5.95	261	16.3125	5.9487	21.46	2006
Guidelines for postoperative care in gynecologic/oncology surgery: Enhanced Recovery After Surgery (ERAS®) Society recommendations–Part II	48	8.49	7.50	196	32.6667	7.5042	24.49	2016
Guidelines for perioperative care in elective rectal/pelvic surgery: Enhanced Recovery After Surgery (ERAS®) Society recommendations	46	4.61	3.55	224	22.4	3.5527	20.54	2012
Fast-track surgery improves postoperative recovery in patients with gastric cancer: a randomized comparison with conventional postoperative care	43	3.61	2.06	112	9.3333	2.056	38.39	2010
A comparison in five European Centres of case mix, clinical management and outcomes following either conventional or fast-track perioperative care in colorectal surgery	42	2.81	1.29	97	5.7059	1.2923	43.30	2005
Determinants of outcome after colorectal resection within an enhanced recovery programme	41	3.55	2.56	120	9.2308	2.5634	34.17	2009

**Table 4:** Most frequent words.

Keywords plus		Author keywords	
Words	Occurrences	Words	Occurrences
care	285	enhanced recovery after surgery	301
metaanalysis	254	eras	159
outcomes	231	enhanced recovery	149
colorectal surgery	222	colorectal surgery	117
perioperative care	209	fast-track surgery	85
complications	200	length of stay	84
resection	183	fast track	71
management	180	perioperative care	70
guidelines	174	laparoscopy	60
program	168	fast-track	52
protocol	141	surgery	49
cancer	122	enhanced recovery after surgery (eras)	46
rehabilitation	115	rehabilitation	38
colonic surgery	111	colorectal cancer	36
impact	104	complications	34
implementation	98	fast track surgery	33
pathway	96	outcomes	31
randomized clinical-trial	96	postoperative complications	31
length-of-stay	83	bariatric surgery	30
surgery	73	colorectal	29
**Titles**		**Abstracts**	
**Words**	**Occurrences**	**Words**	**Occurrences**
surgery	1,363	patients	5,596
recovery	1,092	surgery	3,898
enhanced	981	eras	3,540
patients	316	recovery	2,527
fast_track	241	postoperative	2,454
colorectal	237	stay	1,859
program	226	hospital	1,726
Eras	185	study	1,515
protocol	178	enhanced	1,507
Study	167	days	1,467
pathway	163	care	1,291
cancer	156	protocol	1,254
laparoscopic	153	complications	1,226
undergoing	140	length	1,221
postoperative	124	results	1,169
randomized	122	compared	1,028
implementation	120	outcomes	999
Trial	120	time	969
Care	119	undergoing	950
perioperative	109	significantly	932

### Co-authorship, Co-institute, Co-country and Document Co-citation Analysis

**Figure 4:**
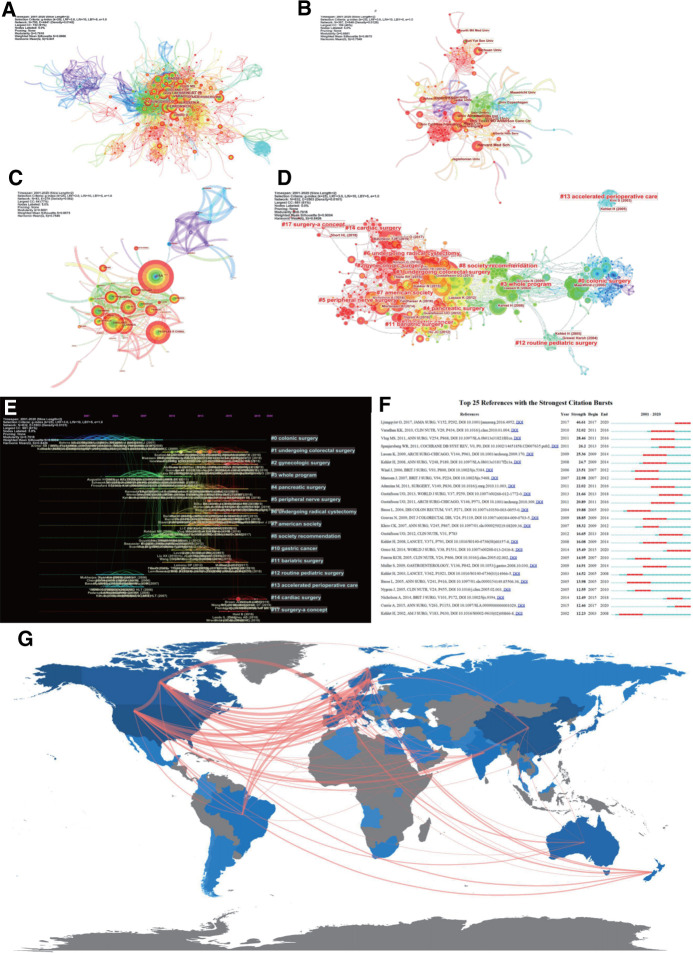
(**A**) An author cooperation map related to ERAS research from 2001 to 2020. (**B**) An institution cooperation map related to ERAS research from 2001 to 2020. (**C**) A country cooperation map related to ERAS research from 2001 to 2020. (**D**) Reference co-citation map related to ERAS research from 2001 to 2020. (**E**) The timeline view of cited reference related to ERAS. (**F**) Top 25 references with the strongest citation bursts. (**G**) The country collaboration map of the global authors of the ERAS research.

The results of the generating collaborator mappings using CiteSpace were 676 nodes and 1,440 links (Figure 4A), which means that 1,401 articles were published by 676 authors. As shown in Figure 4A, many authors preferred to combine with relatively stable collaborators, resulting in a relatively large cluster of authors. In earlier studies, only a few core authors cooperated less with the central clusters. The most representative author was LJUNGQVIST O, who published 33 studies, followed by DEMARTINES N and KEHLET H. The top-ranked item by centrality was O LJUNGQVIST (2005) with a centrality of 30, and the second one was OLLE LJUNGQVIST (2010) with a centrality of 28. The third was H KEHLET (2002) and GREGG NELSON (2016), with centralities of 25.

The institute of the two authors appeared in the same article as a cooperative organization, namely, the co-institute. The Citesapce software was used to calculate the co-occurrence frequency matrix to determine the degree of cooperation. Figure 4B shows the collaborative institutes in the era domain, with an institution map of 367 nodes and 848 links. Generally, the institutions were concentrated in universities and a few in hospitals.

Authors from two different countries appear in the same article; that is, a co-country. Figure 4C shows the results for co-countries in the era domain. As shown in Figure 4C, although the United States was ranks first worldwide in terms of publication volume, European countries had a relatively close cooperative relationship.

After analysis using CiteSpace, Figure 4D shows a document co-citation network diagram containing 832 nodes, 3,503 links, and 15 main clusters. The modularity Q value was 0.7918, and the weighted mean silhouette S was 0.9004.

A literature co-citation analysis of ERAS studies yielded 15 co-citation categories, marked by their citation index terms. To obtain the key cluster of the cited references, log-likelihood tests (LLR) were used to select the noun phrase from the title of the article in Citespace. The contour value of each cluster was >0.9, indicating reliable and meaningful results. Figure 4E summarizes the details of the 15 clusters using a timeline view to reflect the research patterns and emerging trends in the network map. Articles with the strongest citation bursts showed a significant increase in interest in ERAS. Figure 4F shows the 25 strongest references from 2008 to 2020.

Collaborative World Map as a Measure of the Social Structure: There were 709 collaborations between authors from different countries. The world map (Figure 4G and Supplementary material) shows that the most frequent cooperation was between the United States and Canada (24), followed by the United Kingdom and Canada (25), Canada and Sweden, and the United Kingdom and Sweden (23).

### Conceptual Framework and Thematic Evolution

**Figure 5:**
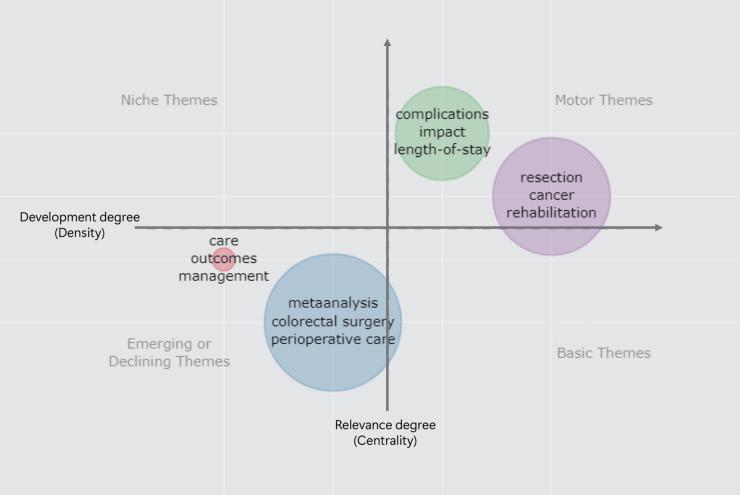
The strategy map of identified topics clustered by keywords plus.

Based on the relationship between the keywords plus, the research content was roughly divided into several topics. The identified topics were categorized into a strategic map to analyze the importance and development of the research topic. The strategy map was based on the full-time span from 2001 to 2020. We used the first 400 keywords, but the items displayed in the cluster were set to a minimum frequency of 40. The number of representative labels in each theme was set to three so that the thematic map based on density (Y-axis) and centrality (X-axis) could be obtained, as shown in Figure 5. The centrality measured the importance of a topic, and density measured the development of the topic. The topics appearing in the lower left were the emerging or declining topics, which are new topics that can emerge better or decline from the research field. The upper right panel represented high density and high centrality. The developed theme of this section was the motor theme, and was necessary.

**Figure 6:**
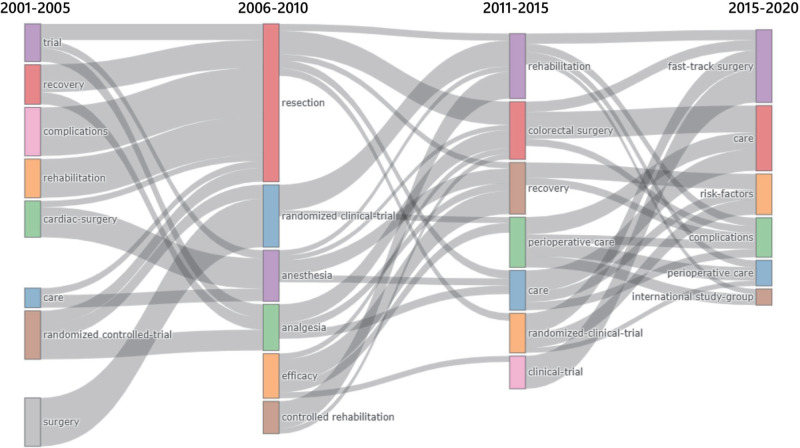
Thematic Evolution of ERAS research from 2001 to 2020.

Thematic evolution showed the historical development of the ERAS literature. The use of keywords and topic evolution described the history and evolution of the topics. This time division was based on the author’s subjective judgment while allowing for a better representation of the evolution of the subject. The first part was from 2001 to 2006, the second from 2007 to 2011, the third from 2012 to 2016, and the last from 2017 to 2021. Figure 6 shows the topic evolution of the keyword plus (Supplementary material).

## Discussion

Fast-track surgery (FTS) or ERAS is the inevitable result of the development of medical theory and technology, “Pain and Risk “Free” was the goal of surgery (26). The connotation of ERAS was to reduce the body’s stress response to trauma, promote rapid functional recovery, reduce the incidence of clinical complications, and shorten the length of hospital stay. A large number of clinical studies have proven that perioperative process optimization and multidisciplinary collaboration of minimally invasive techniques can improve the treatment effects, reduce medical interventions (over-treatment), and promote patient recovery (27). For ERAS to be practiced clinically, its philosophy and associated pathways must be based on evidence-based medicine and multidisciplinary collaboration. It should not only reflect the core concept of accelerating recovery, but should consider the patient’s underlying diseases, types of surgery, perioperative complications, and other specific conditions. Moreover, in-depth clinical studies are required to demonstrate the safety, feasibility, and necessity of ERAS-related pathways.

According to a bibliometric analysis, the United States has the largest number of ERAS-related articles published followed by China. Despite the large number of published reports in China, there were fewer citations. Most of the top 20 most-cited articles were published in the early period of ERAS in Europe and the United States because ERAS originated in Europe and the United States, and were the first to carry out relevant research on ERAS (28, 29). In contrast, as a North American country, Canada was ranks fourth in the total number of articles published; however, the proportion of multi-country publications was high, and most of the articles were from the McGill University, which reflects the high concentration of research. The university with the second highest research focus was the MD Anderson Cancer Center in the United States, which may be due to the fact that the previous studies on ERAS were on radical surgery for patients with tumor.

Earlier researches on ERAS were mainly related to surgery, therefore, the most cited journals in zone 1 of the Bradford’s Law were journals of the surgery discipline, such as the WORLD JOURNAL OF SURGERY, SURGICAL ENDOSCOPY AND OTHER INTERVENTIONAL TECHNIQUES, and COLORECTAL DISEASE. According to the Word Cloud, “colorectal surgery” and “resection” played important roles in surgery and colorectal surgery was the most mature surgical method that used ERAS. With ERAS, the hospital stay and complications in patients who underwent colorectal surgery effectively reduced without an increase in the readmission rates (30).

The ERAS Society was founded in 2001, and in 2005, the first worldwide expert consensus on accelerated recovery for colon resection was developed (29). In addition, relevant studies on ERAS mainly focused on research directions, such as meta-analysis and perioperative care. Randomized controlled trials and meta-analyses have been used in ERAS studies, and a large number of high-quality clinical studies are important for further evidence-based practice and guidelines development. Meta-analysis and clinical research have mainly focused on perioperative nursing, postoperative complications, length of hospital stay, final results, and process management (25, 31). In addition to the ERAS, perioperative care was very important in the implementation to surgical management. Currently, the relevant care specifications are clearly specified in the guidelines for various specialties (7–9). Clinical evidence has shown that perioperative FTS care can promote postoperative rehabilitation and shorten the hospitalization time of patients with gynecological diseases (32). Magheli provided FTS care to 50 patients undergoing laparoscopic radical prostatectomy, which significantly improved the recovery time of the bowel function and defecation time, shortened the postoperative hospital stay, and improved the overall satisfaction rate of patients (33).

Among the 15 clusters obtained through CiteSpace cluster analysis, most studies on ERAS were still related to surgery, but were developed from colorectal surgery in the early stages of cardiac surgery, neurosurgery, and other surgical disciplines. Guidelines are emerging for other general surgery procedures (pancreaticoduodenectomy, elective colon surgery, and elective rectal and pelvic surgery) (10, 11, 24). ERAS has gained acceptance worldwide over time and is widely used in a range of surgical specialties, such as urology, orthopaedics, and obstetrics and gynecology. Although a study on FTS was published in cardiac surgery as early as 1994 (34), it was not until 2015 that Zaouter from France first reported the systematic application of ERAS in cardiac surgery (35). In 2018, Noss systematically reviewed the relevant issues of ERAS in cardiac surgery and provided an in-depth consideration of the existing problems (36). Many urological studies have reported the use of ERAS in the perioperative period of laparoscopic nephrectomy (37), open partial nephrectomy (38), laparoscopic radical prostatectomy (39), and TVT or TVT-O (40). Compared to the control group, the duration of hospital-stay in the patients who underwent ERAS was significantly shorter, with better pain control and patient satisfaction. Similarly, to promote and regulate the use of ERAS in gynecology, the International ERAS Society in 2016 proposed guidelines for the use of ERAS in gynecology/gynecological oncology (41, 42).

After more than 20 years of research, evidence-based medicine demonstrated the effectiveness of ERAS in a rational manner, and ERAS models have demonstrated unprecedented advantages in the recovery of patients undergoing surgery (28). From the thematic maps, studies on complications, impact, length of hospital stay, resection, and cancer were mature, and several of the research directions were supported by evidence-based evidence in the guide. However, research topics such as meta-analyses, colorectal surgery, and perioperative care are declining despite the current large number of studies. Similarly, the thematic evolution of ERAS research in the past 20 years showed that ERAS application and anesthesiology research in tumor surgery were prominent in 2006–2010, while in the next five years, more attention will be paid to the comprehensive management of the perioperative period and the emergence of large randomized controlled clinical trials. Simultaneously, ERAS-related meta-analyses began to appear (43). After 2015, patient-reported outcomes were the purpose of ERAS. More attention has been paid to the comfort and safety of patients in hospitals, as well as the reduction of surgical complications, rather than the reduction of hospital days and cost alone (44–47).

ERAS is not a new technology but an integrated and innovative management mode. Its theoretical system has been relatively well-developed after more than 20 years of development. ERAS concepts and models in different disciplines need to consider the characteristics of their respective disciplines; therefore, there are some differences. Despite the success of the ERAS concept, it still has several challenges. The implementation of the ERAS protocol requires good patient and doctor compliance. A multicenter study found that a reduction in the complications was positively correlated with ERAS compliance (OR = 0.69, *P* < 0.001) (48, 49). At the same time, team cooperation and continuous quality improvement plans are required. The team will formulate the ERAS plan and target management, such as the length of stay, and continue to adhere to and learn the summarized strategies (50). For example, many hospitals in Canada have continuously improved and perfected the clinical practice guideline (CPG) with the application of the “Knowledge-to-Action Cycle” (51), thus slimming the ERAS protocol and increasing the clinical application compliance. Preoperative assessment, preparation, and treatment of patients with high-risk factors and reduction in the failure rate of the ERAS protocols are the major measures to increase patient compliance (52–54). Based on multimodal or multidisciplinary collaboration, the preoperative emphasis on patient education, communication, and collaboration underpins the success of ERAS (55, 56).

Although ERAS has many advantages and is accepted by doctors, there may be limitations to its future use. ① Firstly, the doctors and patients rely on “traditional practices” and “safety considerations.” ② Secondly, from the different systemic conditions, diseases, surgical procedures and hospitals, it was concluded that the ERAS protocols must be “diversified and individualized,” which makes them less evidence-based. ③ Thirdly, the combination of different disciplines may make ERAS processes too convoluted, impeding accelerated recovery. The payment system of medical insurance and cultural background influence the promotion of ERAS programs.

The literature included in this study is from the core collection database of WOS, which has high quality studies, but selection bias cannot be avoided. It is well known that various databases have their advantages and disadvantages, but we chose the WoS over PubMed because PubMed does not store reference metadata, and references were an important part of the research output indicators. In addition, according to the bibliometric research principles, there is a potential length time-lapse bias that puts newer articles at a disadvantage in receiving citations. Of course, bibliometric analyses of the same topic will be used by other authors, and our results may be compared with those of others in the future.

## Conclusion

In this study, Bibliometrix and Citespace were used to analyze ERAS literature over the past 20 years. Despite the rapid development of ERAS in various disciplines, the effective coordination of multidisciplinary physicians and the change in patients’ deeply rooted traditional views were the major obstacles to its further development. The bibliometric analysis conducted in this study is expected to provide a reference for the development of ERAS.

## Data Availability

The original contributions presented in the study are included in the article/Supplementary Material, further inquiries can be directed to the corresponding author/s.

## Data Sharing Statement

The data are available from the authors upon request. Because of the policies of Clarivate, data cannot be shared openly.
